# Crystal Plasticity Modeling of Strain Hardening Induced by Coherent Precipitates in Inconel 718 Superalloy

**DOI:** 10.3390/ma18112436

**Published:** 2025-05-23

**Authors:** Changfeng Wan, Biao Wang

**Affiliations:** School of Material Science and Engineering, Dongguan University of Technology, Dongguan 523808, China; 2024047@dgut.edu.cn

**Keywords:** Inconel 718, crystal plasticity, dislocation–precipitate interaction, strain hardening, geometrically necessary dislocations

## Abstract

In this work, a crystal plasticity (CP)-based continuum modeling approach is employed to investigate the interaction between dislocations and coherent $\gamma^{\prime \prime }$ precipitates in the Inconel 718 (IN718) superalloy. A finite element (FE) model is developed to accurately represent realistic microstructures in IN718, specifically incorporating a disk-shaped precipitate embedded within a matrix phase. A length-scale-dependent CP modeling simulation informed by molecular dynamics (MD) findings is conducted. The results indicate that the three $\gamma^{\prime \prime }$ variants behave differently under uniaxial loading conditions, altering the deformation process in the γ phase and leading to significant strain and stress heterogeneities. The presence of dislocation shearing in the $\gamma^{\prime \prime }$ variants reduces the localization of strain and dislocation densities in the adjacent γ phase. The strain gradient-governed geometrically necessary dislocation (GND) density plays a dominant role in influencing strain hardening behavior. The length scale effect is further quantified by considering four different precipitate sizes, with the major axis ranging from 12.5 nm to 100 nm. The findings show that smaller precipitate sizes result in stronger strain hardening, and the size of $\gamma^{\prime \prime }$ precipitates significantly alters GND density evolution.

## 1. Introduction

IN718, a nickel-based superalloy, is widely utilized in aerospace, petrochemical, and nuclear industries due to its exceptional mechanical properties and outstanding resistance to oxidation and corrosion [1,2,3,4,5,6,7]. This alloy has three main types of precipitates—$\gamma^{\prime }$, $\gamma^{\prime \prime }$, and δ phases—distributed within the γ austenitic matrix [8,9,10]. The γ phase, which forms the matrix, has a face-centered cubic (FCC) structure [11]. The $\gamma^{\prime \prime }$ phase, which serves as the main strengthening component [12], is characterized by an ellipsoidal disc shape with a coherent ${\mathrm{DO}}_{22}$ structure and comprises roughly 15% of the volume [8,9,10]. The tetragonal $\gamma^{\prime \prime }$ phase in IN718 exists in three possible variants, each with distinct 100 habit planes. Although less critical for strengthening, the spherical $\gamma^{\prime }$ precipitate contributes a small volume fraction of about 4% and forms coherently with the structure $L1_{2}$. The needle-like δ phase, found at the grain boundaries, serves to stabilize these boundaries [11,13]. Under external loading, IN718 experiences highly uneven strain and stress at the microstructural level, which drives the strain hardening process and ultimately influences the failure response of the material [14,15]. The stress at the interfaces between the γ and $\gamma^{\prime \prime }$ phases plays a significant role in these processes, as these phases exhibit different plastic behaviors. Therefore, it is essential to mechanistically understand and quantify the influence of precipitates on the resulting strain and stress heterogeneity at the microscale level.

It is well established that $\gamma^{\prime \prime }$ precipitation formation in IN718 contributes to the strengthening of the order and the strengthening of Orowan [8,16,17]. Research by [8,17] demonstrated that when mobile dislocations encounter $\gamma^{\prime \prime }$ precipitates, they tend to shear these precipitates along the 〈110〉 direction, typically as dislocation pairs or quads, leading to order strengthening. Reference [10] observed this dislocation shearing in the form of dislocation pairs and twinning, with the theoretically calculated increment in critical resolved shear stress (CRSS) showing good agreement with experimental data. Additionally, reference [16] observed dislocation loops around fine $\gamma^{\prime \prime }$ precipitates in crept IN718 specimens, suggesting the occurrence of Orowan strengthening, where dislocations bypass the precipitates. The balance between dislocation shearing and bypassing around $\gamma^{\prime \prime }$ precipitates is largely influenced by the size of the precipitates [16,18,19,20,21]. Studies by [10,18,22] indicate that dislocation bypassing becomes the dominant mechanism in the deformation process when the size of $\gamma^{\prime \prime }$ precipitates exceeds a critical threshold. For inelastic deformation within $\gamma^{\prime \prime }$ precipitates, reference [10] suggested that shearing governs deformation in precipitates smaller than 60 nm, while [17] calculated a critical size of approximately 20 nm. Reference [18] later estimated this critical size to be about 12 nm for IN718. In general, both the dislocation shear and bypass mechanisms have been identified for the $\gamma^{\prime \prime }$ phase in IN718, and the relationship between CRSS and the $\gamma^{\prime \prime }$ precipitate size has been quantified. Many experimental studies have been performed to investigate the plasticity behavior on IN718. For example, reference [23] investigated the microstructure and hardness of additively manufactured IN718, and this work provided valuable insights into the microstructural features specific to the additive manufacturing process. The microstructure, which is closely related to precipitate distribution, can affect the hardness of the alloy. Reference [24] employed simulation and experimental methods to analyze the microstructural evolution and mechanical response of laser direct energy deposition IN718. The authors’ research focused on understanding the overall behavior of the alloy during the manufacturing process, providing a basis for understanding the material’s performance and its precipitate-related aspects. Reference [25] studied the deformation behavior of selective laser melted IN718. The authors’ work focused on how the alloy deforms under various conditions. Although it has made significant contributions in many aspects related to the hardening behavior, there remains a notable absence of integration between the observed deformation behavior and the underlying precipitate-hardening mechanisms. A comprehensive understanding of strain hardening due to coherent $\gamma^{\prime \prime }$ precipitates, particularly regarding their load-carrying capacity, slip mechanism interactions, dislocation–precipitate interactions, and the size effect of $\gamma^{\prime \prime }$ precipitates in IN718, remains incomplete.

Significant efforts have been devoted to quantifying the role of nano-precipitates during inelastic deformation in metallic materials. MD simulations have emerged as an ideal tool for tracking dislocation motions and explicitly accounting for the effects of nanoscale precipitates [26,27,28,29,30,31]. For example, reference [26] investigated the interaction between edge dislocations and copper precipitates in α-iron alloys, quantifying the dependence of CRSS on temperature, precipitate size and mismatch level. The findings suggested that smaller precipitate sizes and/or higher temperatures promote dislocation shearing. Similar trends have been observed in other alloy systems, including medium- and high-entropy alloys [27,28,30], Mg-Al alloys [29], and Mg-Zn alloys [31]. Furthermore, MD simulations have been used to examine how plastic flow and dislocation motion are influenced by the size of nanopores or nanoparticles, as seen in studies on copper [32]. On the other hand, continuum modeling approaches, particularly those based on crystal plasticity theory, have been extensively developed to investigate the mechanical behavior of precipitate-strengthened alloys at the microstructural level [13,33,34,35,36,37,38,39,40]. Crystal plasticity models are designed to describe plastic deformation by considering the slip systems within individual grains. These models have been applied to the Inconel 718 superalloy to study various aspects of its mechanical behavior. For example, references [35,41,42] developed a self-consistent mean field model that introduces internal variables in the constitutive laws to account for the effects of precipitate size and volume fraction. Reference [20] advanced a full-field elasto-viscoplastic model using a fast Fourier transform formulation to simulate plastic deformation in IN718, showing that the size of the precipitate significantly affects the resistance to crystallographic slip within the crystal plasticity framework. Similarly, reference [37] proposed a crystal plasticity model that simulates the stress–strain response of IN718 under both cyclic and monotonic loading conditions, incorporating the effects of precipitate size, spacing, and the evolution of back stress and CRSS. Reference [18] combined a crystal plasticity model with the Lifshitz–Slyozov–Wagner law to account for precipitate strengthening during the heat treatment of IN718. However, while these continuum models have successfully captured certain aspects of precipitate behavior in IN718, they often lack a detailed consideration of how precipitate morphology affects slip behavior. Despite the success of continuum models in other materials, such as martensitic steels [43] and single crystal nickel-based superalloys [33], a more complete understanding of the microplasticity development and stress partitioning caused by precipitates in IN718 is still needed.

Despite progress in both precipitate hardening research and crystal plasticity modeling, there is a substantial gap between the two. Research on precipitate hardening in the Inconel 718 alloy has predominantly been experimental. These studies focus on observing the microstructure and measuring mechanical properties to elucidate the strengthening mechanisms associated with precipitate formation [6,12]. Reference [44] examined the effect of a gradient structure on the mechanical performance of IN718 at elevated temperatures. While this work was more centered on the gradient structure, it is relevant as the precipitate morphology can be affected by the gradient, and understanding this could be beneficial for integrating with crystal plasticity models. In contrast, crystal plasticity models, while effective in predicting the overall plastic deformation of the alloy, often simplify the complex interactions between precipitates and the matrix. For example, in crystal plasticity models, the presence of precipitates, which are known to impede dislocation movement, a fundamental process in plastic deformation, is not fully accounted for. Although models have been developed to study the tensile anisotropic mechanisms in the additive manufactured IN718 alloy [39], they lack detailed consideration of how precipitate-induced strengthening influences the observed anisotropic behavior. The orientation of grains and precipitates plays a crucial role in the alloy’s anisotropy, but current crystal plasticity models do not accurately capture the intricate relationship between precipitate hardening and anisotropic mechanical properties. Similarly, in the quantification of the anisotropic tensile and fatigue properties of the laser powder bed using crystal plasticity [45], the impact of precipitate hardening on these anisotropic behaviors remains under exploration. Bridging this gap between precipitate hardening and crystal plasticity modeling is of utmost importance. It will not only enhance our fundamental understanding of the mechanical behavior of the Inconel 718 alloy but also offer more precise guidance for material design and optimization of manufacturing processes in practical applications.

Overall, recent studies do not fully address the competition between dislocation shearing and bypassing mechanisms. Therefore, it is crucial to quantify the influence of precipitates on mechanical response, stress–strain evolution, dislocation density distribution, dislocation–precipitate interactions, and stress partitioning between the matrix and precipitates using crystal plasticity methods.

This research builds upon the foundational work of [46], which established the primary principles in the interaction between precipitate and dislocation in IN718. Such interaction has been proved to have a deep influence on the hardening behavior [47,48]. The study demonstrated the follow findings: (1) the slip system is activated preferentially in the precipitate, (2) the flow stress is lower in the matrix than in the precipitate, and (3) the dislocations propagate faster in the matrix than in the precipitate. These findings serves as a basis for the current investigation. While the previous work [46] provided substantial insights into these certain aspects, the mechanical behavior on the continuum level remains unexplored. In this study, the crystal plasticity method is applied to further explore this. The crystal plasticity modeling study is performed to quantify the strain hardening produced by $\gamma^{\prime \prime }$ precipitates in IN718. The objectives of this work are to (i) elucidate the strengthening mechanisms with respect to shearing and bypassing in IN718 and (ii) quantify how precipitate–dislocation interactions affect the mechanistic response and stress partition in the material.

This paper is structured as follows. Section 2 and Section 3 presents the characterization and the CP models. Section 4 outlines the results, which are discussed in Section 5, followed by concluding remarks in Section 6.

## 2. Material Preparation and Microstructural Characterization

**Figure 1:**
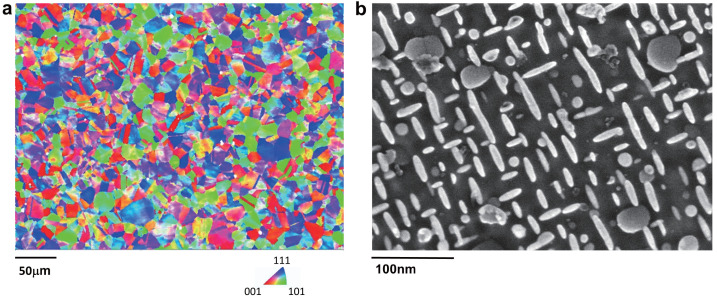
Microstructural characterization for IN718 with (**a**) EBSD measurement and (**b**) TEM characterization.

The IN718 superalloy investigated in this study was fabricated using standard melting processes, followed by heat treatment and forging [49,50]. Table 1 presents the chemical composition of the superalloy, as received. Electron back scatter diffraction (EBSD) measurements were conducted to characterize the microstructure, as depicted in Figure 1a. The EBSD scanning area was $800\times 600\;{\mathrm{mm}}^{2}$, with a step size of 1.5μm. As can be observed from Figure 1a, the texture exhibits a quasirandom distribution, and the average grain size is approximately 20μm. Transmission electron microscopy (TEM) was also used to examine the precipitate structure within a grain, as shown in Figure 1b. Disc-like ellipsoidal $\gamma^{\prime \prime }$ precipitates are clearly visible in Figure 1b, with the average lengths of the major and minor axes of these $\gamma^{\prime \prime }$ precipitates being approximately 25nm and 12nm.

## 3. Modeling Methods

### 3.1. Crystal Plasticity-Based Micromechanical Finite Element Models

**Figure 2:**
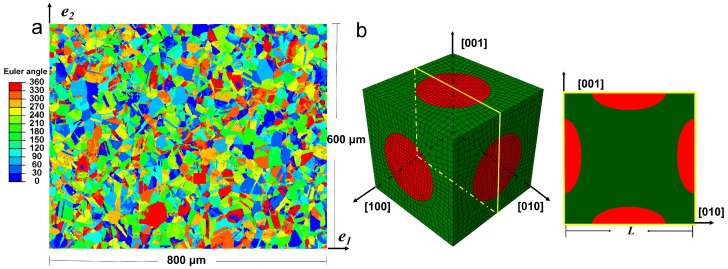
The finite element models at different length scales: (**a**) the Euler angle distribution of the representative volume element at the mesoscale and (**b**) isometric and midplane-section views of the unit cell model at the microscale.

To obtain the constitutive response at the grain level, a two-scale finite element modeling approach is used. First, a FE model is developed representing the realistic polycrystalline microstructure of IN718 based on EBSD measurements. Similarly to the work by [43,51], the FE model is assigned Euler angle information directly corresponding to the EBSD data. Figure 2a illustrates the distribution of one of the three proper Euler angles within the polycrystal FE model. These Euler angles are generated through intrinsic rotations about the axes [100], [100], and [001]. The constitutive response of each grain is governed by a crystal plasticity model, which will be detailed in a subsequent section. Three-dimensional finite elements are used, with the mesh in the normal (out-of-plane) direction consisting of only one element, which is sufficient for plane strain loading conditions. In this study, uniaxial loading is applied along the $e_{1}$ direction, allowing the polycrystal model to predict the macroscopic response of IN718. Secondly, a unit cell model is developed to represent the explicit morphological features at the grain level, specifically the $\gamma^{\prime \prime }$ precipitate and γ matrix. Figure 2b depicts both the surface and the interior of the FE model, showcasing it from an isometric view as well as a midplane-section view. The following simulations will utilize these two distinct viewpoints to display the outcomes. The FE model includes three disk-shaped $\gamma^{\prime \prime }$ variants, with the *c*-axis aligned along the [100], [010], and [001] directions. The edge length of the unit cell, *L*, is 43 nm, while the major and minor axes of the ellipsoidal variant measure 25 and 12 nm, respectively. Unless otherwise specified, the subsequent simulations will maintain these dimensions. The volume fraction of the $\gamma^{\prime \prime }$ phase is approximately 15% and remains constant throughout the study. Periodic boundary conditions are applied to both the polycrystal model and the unit cell model. The unit cell model can be considered as a representative material point from the polycrystalline model. Uniaxial tensile loading is applied to the unit cell model along the [010] direction, effectively simulating uniaxial loading along the 〈100〉 direction of a single IN718 crystal. The strain rate of all the following simulation is $10^{-3}$ $s^{-1}$.

### 3.2. Constitutive Formulation

In the microstructure-based FE models described above, strain gradient-based crystal plasticity theory is employed to capture the material’s inelastic constitutive behavior. Within this framework, inelastic slip is permitted on twelve 110 [111] slip systems, which are representative of the homogenized FCC crystal structure in both the polycrystal FE model and the γ and $\gamma^{\prime \prime }$ phases in the unit cell model. This approach allows for a detailed and accurate representation of the inelastic deformation mechanisms at the microstructural level.

Following the work of [52], the deformation gradient, F, can be multiplicatively decomposed into elastic and plastic components:

(1) $$F=F^{e}F^{p},$$

the elastic part, $F^{e}$, represents both the elastic stretch and rigid body rotation of the material, while the plastic part, $F^{p}$, describes the pure plastic deformation, which primarily includes dislocation slip and other irreversible deformations. This separation allows for a clear distinction between the reversible and irreversible components of the overall deformation, enabling a more accurate description of the material’s behavior under various loading conditions. Following the work of [53], the plastic velocity gradient for crystalline materials, $L^{p}$, is written as,

(2) $$L^{p}={\dot{F}}^{p}{\left(F^{p}\right)}^{-1}=\sum_{\alpha =1}^{12}{\dot{\gamma }}^{\alpha }m^{\alpha }⊗n^{\alpha },$$

where ${\dot{\gamma }}^{\alpha }$ is the slip rate of the αth slip system, $m^{\alpha }$ and $n^{\alpha }$ are the unit vectors of the slip direction and the slip plane normal, respectively, for the αth slip system, and ⊗ represents a dyadic product.

The following linear elastic constitutive relationship holds between the work-conjugate pair, the second Piola–Kirchhoff stress in the intermediate configuration, $T^{*}$, and the elastic Green strain, $E^{e}$ (see, e.g., [33,54]),

(3) $$T^{*}=C:E^{e},$$

where C is the elastic stiffness tensor and : represents the tensorial double contraction product. The stress tensor, $T^{*}$, is written as,

(4) $$T^{*}={\left(F^{e}\right)}^{-1}J\sigma {\left(F^{e}\right)}^{-T},$$

where J=det(F) and σ is the Cauchy stress.

The elastic Green strain tensor, $E^{e}$, is written as,

(5) $$E^{e}=\frac{1}{2}\left({\left(F^{e}\right)}^{T}F^{e}-I\right),$$

where I is the second-order identity tensor.

For the crystallographic slip deformation on the αth slip system, the following exponential type of constitutive relationship (flow rule) is used for the work-conjugate pair, the resolved shear stress, $\tau^{\alpha }$, and the slip rate, ${\dot{\gamma }}^{\alpha }$ (see, e.g., [34,55]),

(6) $${\dot{\gamma }}^{\alpha }={\dot{\gamma }}_{0}\exp\left\{-\frac{F}{kT}{\langle 1-{\langle \frac{|\tau^{\alpha }|-S^{\alpha }}{\tau_{0}}\rangle }^{p}\rangle }^{q}\right\}\mathrm{sgn}\left(\tau^{\alpha }\right),$$

where *F*, *k*, and *T* are the thermal activation energy, Boltzmann constant, and absolute temperature, respectively; *p* and *q* are the exponents constants; ${\dot{\gamma }}_{0}$ is the pre-exponential constant; $\tau_{0}$ is the lattice friction stress at 0 K; and $\tau^{\alpha }$ and $S^{\alpha }$ are the resolved shear stress and slip resistance on the αth slip system. The resolved shear stress, $\tau^{\alpha }$, is written as (see, e.g., [33,54]),

(7) $$\tau^{\alpha }={F^{e}}^{T}F^{e}T^{*}:\left(m^{\alpha }⊗n^{\alpha }\right).$$

The constitutive laws described above incorporate the effects of evolving slip barriers during plastic deformation by introducing the internal variable $S^{\alpha }$. To relate slip resistance to the material’s microstructural state, a generalized Taylor relation [56] is employed. This relation connects slip resistance to both the statistically stored dislocation (SSD) density and the geometrically necessary dislocation (GND) density, thereby providing a comprehensive description of the material’s hardening behavior as it undergoes plastic deformation. This approach allows for a more accurate prediction of how the material’s resistance to slip evolves as dislocations accumulate and interact with each other.

(8) $$S^{\alpha }=\mu b\sqrt{\sum_{\beta =1}^{12}h_{S}^{\alpha \beta }\rho_{S}^{\beta }+\sum_{\beta =1}^{12}h_{G}^{\alpha \beta }\rho_{G}^{\beta }},$$

where μ and *b* are the shear modulus and the magnitude of Burgers vector, respectively; $\rho_{S}^{\beta }$ and $\rho_{G}^{\beta }$ represent SSD density and GND density on the βth slip system, respectively; and $h^{\alpha \beta }$ is the interaction matrix for the dislocation densities. The matrix is usually expressed as,

(9) $$h_{i}^{\alpha \beta }=h_{0}\left[\omega_{1i}+\left(1-\omega_{2i}\right)\delta^{\alpha \beta }\right],i=S,G,$$

where $h_{0}$ is a coefficient representing the interaction intensity, $\omega_{1}$ and $\omega_{2}$ are constants, and $\delta^{\alpha \beta }$ is the Kronecker delta. The SSD density can be decomposed into the edge and screw parts as follows,

(10) $$\rho_{S}^{\alpha }=\rho_{S_{e}}^{\alpha }+\rho_{S_{s}}^{\alpha }.$$

where $\rho_{S_{e}}^{\alpha }$ and $\rho_{S_{s}}^{\alpha }$ are the edge and screw parts, respectively. The rates of $\rho_{S_{e}}^{\alpha }$ and $\rho_{S_{s}}^{\alpha }$ are governed by [43,56],

(11) $${\dot{\rho }}_{S_{e}}^{\alpha }=\frac{1}{b}\left[\frac{1}{Y_{e}^{\alpha }}-d_{e}\rho_{S_{e}}^{\alpha }\right]\left|{\dot{\gamma }}^{\alpha }\right|,$$

(12) $${\dot{\rho }}_{S_{s}}^{\alpha }=\frac{1}{b}\left[\frac{1}{Y_{s}^{\alpha }}-\left(1+\frac{\pi d_{s}}{4Y_{s}^{\alpha }}\right)d_{s}\rho_{S_{s}}^{\alpha }\right]\left|{\dot{\gamma }}^{\alpha }\right|,$$

where $Y_{e}^{\alpha }$, $Y_{s}^{\alpha }$ are the mean free paths of the edge and screw dislocations, respectively, and $d_{e}$, $d_{s}$ are the critical annihilation distances of the edge and screw dislocations, respectively. Similar to [43], $Y_{e}^{\alpha }$, $Y_{s}^{\alpha }$ are assumed to be inversely proportional to the slip resistance,

(13) $$\frac{1}{Y_{j}^{\alpha }}=\frac{K_{j}S^{\alpha }}{\mu b},j=e,s,$$

where $K_{j}$ is a coefficient depending on the dislocation type.

Following the work of [56], the GND density can be decomposed into three parts as follows,

(14) $$\rho_{G}^{\alpha }=\left|\rho_{G_{sm}}^{\alpha }\right|+\left|\rho_{G_{et}}^{\alpha }\right|+\left|\rho_{G_{en}}^{\alpha }\right|,$$

where $\rho_{G_{sm}}^{\alpha }$ is the pure screw dislocation component in the slip direction, $m^{\alpha }$, and $\rho_{G_{et}}^{\alpha }$ and $\rho_{G_{en}}^{\alpha }$ are the pure edge components in the directions $t^{\alpha }$ ($t^{\alpha }=n^{\alpha }\times m^{\alpha }$) and $n^{\alpha }$, respectively. Then, the rate of the vectorized GND density depends on the slip rate and the plastic deformation gradient as follows,

(15) $${\dot{\rho }}_{G_{sm}}^{\alpha }m^{\alpha }+{\dot{\rho }}_{G_{et}}^{\alpha }t^{\alpha }+{\dot{\rho }}_{G_{en}}^{\alpha }n^{\alpha }=\frac{1}{b}\mathrm{curl}\left(\dot{\gamma^{\alpha }}n^{\alpha }F^{p}\right).$$

The accumulated equivalent plastic strain is used to describe the local plastic deformation [43], and it is written as,

(16) $$\varepsilon_{eq}=\int_{0}^{t}{\left(\frac{2}{3}D^{p}:D^{p}\right)}^{\frac{1}{2}}d\tau ,$$

where *t* is the current time, τ is the integration variable of time, and $D^{p}$ is the plastic strain rate, defined as,

(17) $$D^{p}=\frac{1}{2}\sum_{\alpha =1}^{12}{\dot{\gamma }}^{\alpha }\left[F^{e}m^{\alpha }⊗n^{\alpha }{\left(F^{e}\right)}^{-1}+{\left(F^{e}\right)}^{-T}n^{\alpha }⊗m^{\alpha }{\left(F^{e}\right)}^{T}\right].$$

For the numerical implementation of the presented strain gradient crystal plasticity model, a user-defined element (UEL) has been developed within a commercial FE package. Further details of the UEL formulation can be found in the work of [43,56].

### 3.3. Calibration of the CPFE Model Assisted by Experimental Data and MD Simulations

Due to the absence of single-crystal stress–strain data for IN718, the following strategy is adopted to calibrate the unit cell model. First, the crystal plasticity method is applied to the polycrystal. The parameters are then calibrated to ensure that the results match the experimental data. Subsequently, a finite element simulation in one element is performed using the same parameters. The simulation results obtained with one element can be considered as homogeneous data in a single crystal. To calibrate the unit cell model, the parameters are adjusted once again and the stress–strain data of the unit cell are compared with the data from the one element. During this second calibration process, only a few parameters are modified, and these changes are kept within a reasonable range. The details of the changes of the parameters in both the matrix phase and the precipitate phase are presented below.

In the proposed crystal plasticity-based FE models for both the polycrystal and the unit cell, three distinct materials are involved: the γ phase and the $\gamma^{\prime \prime }$ phase in the unit cell model, and the homogenized crystal in the polycrystal model. Each material is characterized by 18 material parameters. Given that the γ and $\gamma^{\prime \prime }$ phases are coherent and the primary focus of this study is on strain hardening during plastic deformation, the elastic constants for these two phases are assumed to be identical. Consequently, all three materials in the model share the same elastic constants. These constants are determined from the diffraction elastic constants of IN718, as measured by in situ neutron diffraction tests [57], and are used to derive the single crystal elastic constants [58], as detailed in Table 2.

The table also lists the parameters used in the flow rule and hardening law for the homogenized crystalline phase. According to [59], the values of the exponential coefficients, *p* and *q*, can capture the trends observed in atomistic data. A relatively large value for $\dot{\gamma_{0}}$ is chosen to appropriately represent strain rates under quasi-static conditions [60]. The value of *F* used here is taken from [33] for the CMSX4 superalloy. The parameter $\tau_{0}$, which corresponds to the yield strength of the material, is calibrated by fitting the model’s stress–strain predictions to experimental data.

For the strain hardening parameters, the Burgers vector for IN718 is sourced from [35,61]. It has been suggested [56,62] that $K_{s}$ is twice $K_{e}$, and $d_{s}$ is ten times $d_{e}$; these ratios are adopted in the present work. Taylor hardening is assumed for the evolution of the SSD density, while self-hardening is applied for the evolution of the GND density. The values of $h_{0}$, $K_{e}$, and $d_{e}$ are influential in determining the strain hardening response and are calibrated by fitting the simulated stress–strain curves to experimental data with the method of Least Squares. The initial total dislocation density is set to $1.5\times 10^{6}\;{\mathrm{mm}}^{-2}$, as reported in [35], with the initial dislocations assumed to be entirely SSD.

**Figure 3:**
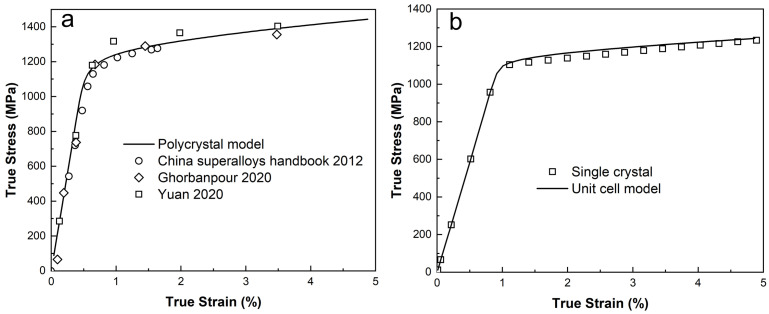
Calibration of (**a**) polycrystal model with macroscale stress–strain data for IN718 at room temperature [13,42,63] and (**b**) unit cell model with nanoscale stress–strain data for IN718 single crystal with loading along [010] direction.

In the current unit cell model (Figure 3b), both the γ and $\gamma^{\prime \prime }$ phases are capable of sustaining plastic deformation. Based on findings from MD simulations [46], each variant of the $\gamma^{\prime \prime }$ phase has four active slip systems. Specifically, for the [100] variant, the A2, B2, C1, and D1 octahedral slip systems are active. The [010] variant activates the A3, B4, C3, and D4 slip systems, while the [001] variant utilizes the A6, B5, C5, and D6 slip systems. Another important insight from the atomistic simulations is that dislocation movement is more difficult in the precipitate than in the matrix. As a result, adjustments to the material parameters for the γ and $\gamma^{\prime \prime }$ phases in the unit cell FE model are necessary.

Table 3 presents the modified values of the relevant parameters. The parameter $K_{e}$ is physically linked to the dislocation mean-free path. Given the coherency between the γ and $\gamma^{\prime \prime }$ phases, it is assumed that both phases share the same value of $K_{e}$. The value of $K_{e}$ can be calibrated by fitting the aggregate stress–strain data from the unit cell model to the stress–strain response of the homogenized phase, which can be computed using a single-element FE model with the constitutive parameters listed in Table 2.

The parameter $h_{0}$ reflects the slip resistance in the constitutive model and is closely related to the overall yield strength. To remain consistent with the atomic modeling results, the $\gamma^{\prime \prime }$ phase is assigned higher values of $h_{0}$ and $\tau_{0}$ compared with the γ phase. These values for the $\gamma^{\prime \prime }$ phase are also calibrated using a similar procedure as for $K_{e}$. For simplicity, it is assumed that the matrix γ phase has the same values of $h_{0}$ and $\tau_{0}$ as the homogenized phase.

Figure 3 presents the stress–strain data at room temperature from both experiments [13,42,63] and simulations. In Figure 3a, the simulated stress–strain curve based on the polycrystalline FE model shows good agreement with the experimental measurements, reflecting the effectiveness of material parameter calibration. The calibrated crystal plasticity model at the mesoscale, which represents the homogenized crystal, is then employed to simulate the stress–strain response of a single crystal under uniaxial loading along the [010] direction, as depicted by the open square symbols in Figure 3b. Based on these data, the constitutive parameters for the γ and $\gamma^{\prime \prime }$ phases are calibrated in the unit cell FE model.

## 4. Results

### 4.1. Multiscale Stress–Strain Response

**Figure 4:**
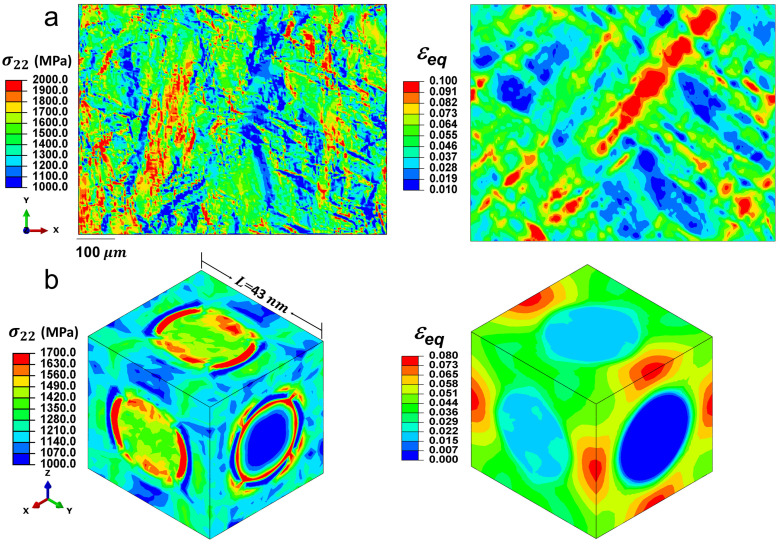
Distributions of the stress along the loading direction and the accumulated equivalent plastic strain from (**a**) the polycrystal model and (**b**) the unit cell model. The tensile strain is 5% in both cases.

Figure 4 illustrates the strain and stress distributions at different length scales, simulated using both the polycrystal FE model and the unit cell model at 5% tensile strain. In Figure 4a, strong strain and stress localizations are evident. The local stress along the tensile direction varies significantly, ranging from approximately 1000 MPa to 2000 MPa, while the average stress of the polycrystalline aggregate is around 1400 MPa (as shown in Figure 3a). At 5% applied strain, the local accumulated equivalent plastic strain in the polycrystal can reach up to 10%. These pronounced heterogeneities in stress and strain are attributed to the polycrystalline nature of the alloy, which consists of ’hard’ and ’soft’ grains that respond differently to elastic and plastic deformations. The realistic crystallographic orientations used in the current FE model effectively capture these polycrystalline characteristics.

In Figure 4b, the results from the unit cell model indicate that stress localization along the loading direction primarily occurs at the phase boundaries, or interfaces, where there is a significant stress gradient. Additionally, the [100] and [001] variants are observed to sustain higher stress levels along the loading direction compared with the matrix phase, with maximum stress reaching up to 1700 MPa and minimum stress remaining above 1300 MPa. The stress distribution in the [010] variant differs from that in the [100] and [001] variants, primarily because the short axis of the [010] variant is aligned with the loading direction, whereas the long axes of the [100] and [001] variants are parallel to the loading direction, leading to different stress distributions within the variants. The ratio of stress between the variants and the overall model, referred to as stress partitioning, has been carefully examined.

The accumulated equivalent plastic strain distribution is also presented in Figure 4b. The [010] variant behaves like an elastic particle, accumulating nearly zero plastic strain, while the [100] and [001] variants undergo plastic deformation, accumulating approximately 2% plastic strain. This behavior is expected, as each variant in the current unit cell model allows for four active slip systems, and the resolved shear stress in the [010] variant is insufficient to activate these slip systems. Another notable feature of the accumulated equivalent plastic strain is that strain localization tends to occur in the regions between the [010] variant and the [100] or [001] variants, with strain levels reaching up to 8%.

The stress partitions along the loading direction in the three variants are further analyzed. Stress partition, in this context, is defined as the ratio of the integral of stress within a variant to the total stress in the matrix. This metric is crucial for constructing diffraction patterns in neutron diffraction [57], particularly because it is challenging to distinguish the $\gamma^{\prime \prime }$ phase peak from the matrix in these patterns.

**Figure 5:**
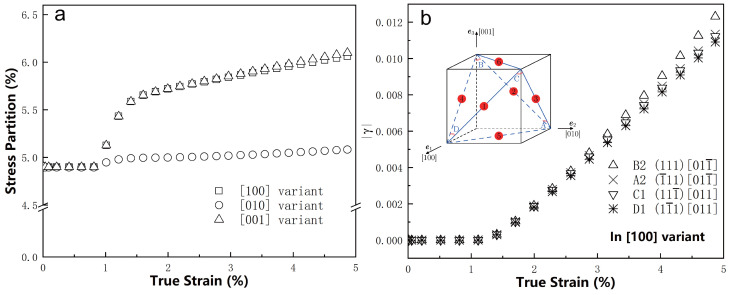
Stress and strain evolution of the variants during the deformation, (**a**) stress partitioning in three variants and (**b**) crystallographic shear development of the [100] variant.

Figure 5a compares the stress partitions across the three variants at different applied strains. The stress partition here is calculated as the fraction of the volume-average stress along the loading direction within a variant over the overall volume-average stress in the unit cell along the same direction. When the applied strain is below approximately 1% (where the unit cell deforms elastically), all three variants sustain similar stress levels, each with a stress partition of about 5%. This uniformity occurs because the γ and $\gamma^{\prime \prime }$ phases share the same elastic constants in the model.

As the applied strain increases beyond 1% and the unit cell begins to deform plastically, the stress partition of the [010] variant remains relatively unchanged from its value in the elastic regime. However, the stress partitions for the [100] and [001] variants gradually increase, reaching up to around 6%. This increase is attributed to the incompatible plastic deformation between the γ phase and the [100] and [001] variants, which leads to the development of inter-phase stresses within these two variants.

To further investigate the plastic strain evolution in these variants, Figure 5b presents the development of plastic slip in the four slip systems of the [100] variant. Due to the geometrical symmetry between the [100] and [001] variants, only the [100] variant is analyzed here. The figure shows the volume-average plastic slip for this variant plotted against the applied true strain of the unit cell. When the applied strain is below 1%, the shear slip is negligible. However, as the strain increases beyond 1%, the plastic slip in all four slip systems rises sharply. Among these, the B2 slip system of the [100] variant exhibits the largest plastic slip. This variation in response may be related to the uneven distribution of plasticity within the variant, as observed in Figure 4b.

### 4.2. The Distribution and Evolution of the SSD and GND Dislocation Density

**Figure 6:**
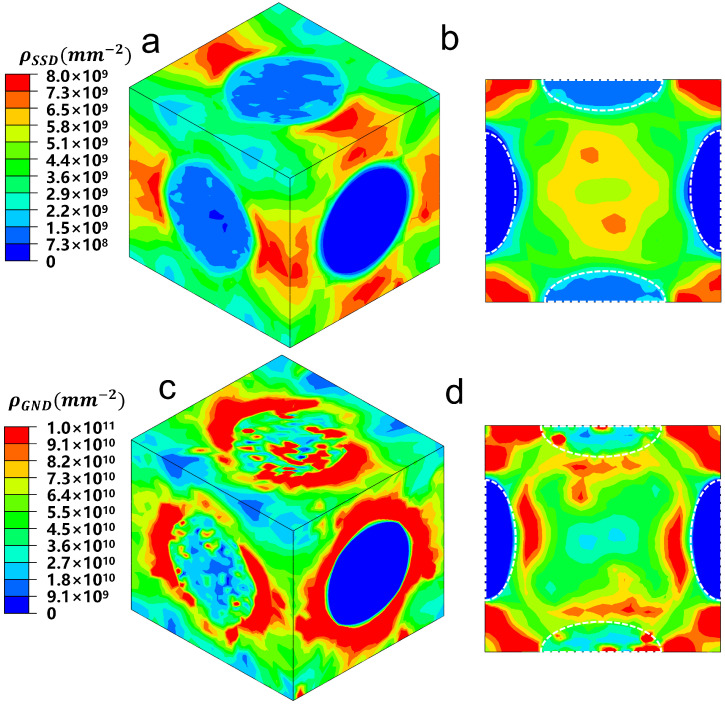
Dislocation density distributions simulated by the unit cell model with (**a**) SSD density distribution at 5% applied strain, (**b**) the cross-sectional view of SSD density distribution, (**c**) GND density distribution at 5% applied strain, and (**d**) the cross-sectional view of GND density distribution.

The dislocation density distribution and evolution within the unit cell are key to understanding the physical origins of strain hardening induced by precipitates. Figure 6 displays the distributions of SSD and GND as simulated by the unit cell model at 5% applied true strain. The figure reveals distinct patterns for the two types of dislocation densities.

The SSDs tend to accumulate primarily in the regions between the [010] variant and the [100] and [001] variants. This accumulation occurs because the [100] and [001] variants are capable of accommodating plastic deformation during loading, allowing a certain level of SSD density to build up within these variants. In contrast, the GNDs tend to accumulate near the precipitates, particularly around the [010] variant, due to the large plastic strain gradients that typically form near these precipitates. Since the [010] variant does not undergo plastic deformation, dislocations tend to surround it, consistent with the Orowan loop mechanism [64].

For the [100] and [001] variants, fewer dislocations accumulate nearby, as the dislocations are able to cut through these variants. Nonetheless, similar to the SSDs, a considerable number of GNDs can still be observed within the [100] and [001] variants. Notably, the localized GND density is approximately an order of magnitude higher than the SSD density, highlighting the significant role of GNDs in contributing to the overall strain hardening observed in the material.

**Figure 7:**
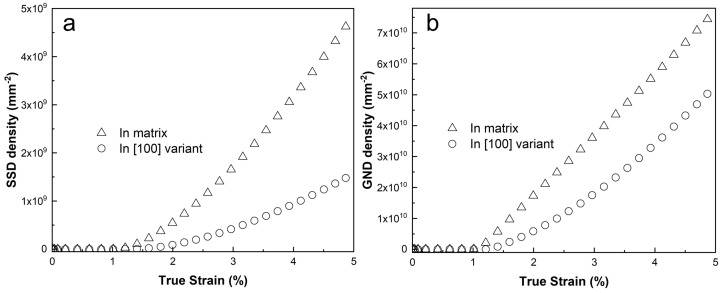
Dislocation density evolution in matrix and precipitate during elastic and plastic deformation.

Figure 7 illustrates the evolution of volume-average SSD and GND densities across all elements in the matrix and the [100] variant. The figure shows that both GND and SSD densities remain constant while the material is in the elastic regime and then gradually increase as the applied strain enters the plastic regime.

When the unit cell begins to deform plastically, the matrix phase exhibits a much higher SSD density compared with the [100] variant. This is because the matrix has more active slip systems than the [100] variant, allowing for greater dislocation activity and, consequently, higher SSD accumulation. On the other hand, the GND densities in the matrix and the [100] variant are found to be at similar levels. This is expected, as the evolution of GND density is influenced not only by plastic slip but also by the strain gradient, as described by Equation (15). Therefore, even though the [100] variant has fewer active slip systems, the strain gradients in this variant contribute significantly to the generation of GNDs, leading to comparable GND densities in both the matrix and the [100] variant.

### 4.3. Dislocation Shearing Effect

Based on the MD simulation results [46], the $\gamma^{\prime \prime }$ precipitate tends to activate four slip systems on which dislocation shearing can occur. To investigate the effect of dislocation shearing, which is presumably a key strain hardening mechanism produced by coherent nano-precipitates, a comparative study was conducted. An additional simulation was performed using the unit cell model, with all three variants assumed to behave as elastic particles (representing the unshearable case).

**Figure 8:**
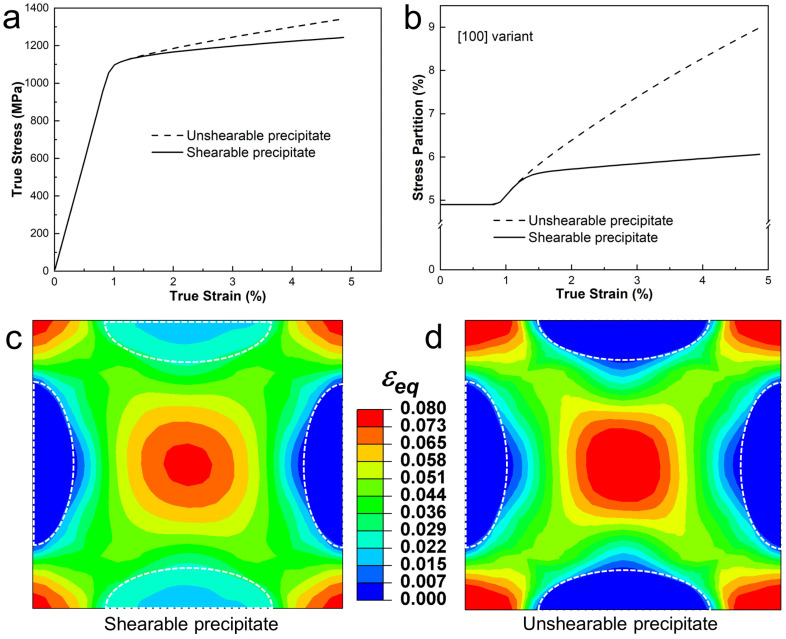
Shearing effect in terms of (**a**) stress–strain prediction and (**b**) stress partition evolution is investigated, and the equivalent accumulate plastic strain distribution under 5% true strain is showed in unit cell model with (**c**) plastic precipitates and (**d**) elastic precipitates.

Figure 8a compares the simulated overall stress–strain curves for the cases with shearable and unshearable variants. The results indicate that allowing dislocation shearing reduces the extent of strain hardening, as it effectively softens the variants. The stress partition trends in the [100] variant, with and without the shearing effect, are shown in Figure 8b. When the unit cell deforms plastically, the stress partition in the shearable variant is significantly higher than in the unshearable variant, indicating a strong shearing effect. A similar trend is observed for the [001] variant due to the geometrical symmetry between the [100] and [001] variants. In contrast, no significant effect is seen for the [010] variant, as it behaves like an elastic particle under the current loading conditions, regardless of whether it is shearable or not. The stress partition trends in the [001] and [010] variants are not presented here for the interests of space.

Figure 8c,d show the simulated distributions of the accumulated equivalent plastic strain (midplane view) at 5% applied strain for the unit cell models with and without dislocation shearing, respectively. Both models reveal that the regions between the $\gamma^{\prime \prime }$ variants undergo significant plastic deformation with strong strain localization. However, the model with dislocation shearing predicts weaker strain localization compared with the model without the shearing effect, highlighting the impact of dislocation shearing on reducing strain localization.

### 4.4. Length-Scale Dependence of the Unit Cell Model

**Figure 9:**
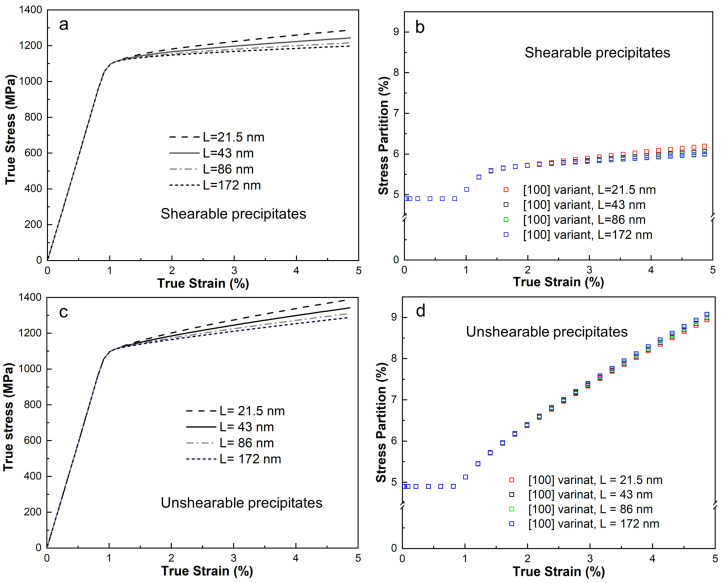
Stress–strain prediction in unit cell model with various model sizes with (**a**) and without (**c**) shearing effect and (**b**,**d**) their corresponding stress partition evolution.

In nickel-based superalloys, the size of precipitates is a key factor influencing strain hardening [65]. Therefore, understanding how the unit cell model depends on length scale is of practical interest. Four different unit cells, with edge lengths ranging from 21.5 nm to 172 nm, corresponding to $\gamma^{\prime \prime }$ precipitates with major axis lengths from 12.5 nm to 100 nm, were subsequently examined. These unit cells are geometrically identical in a dimensionless sense. Since dislocation shearing and bypassing are closely tied to precipitate size, simulations were performed under two conditions: with shearable and unshearable precipitates for all unit cells. Figure 9a,b compare the stress–strain curves and stress partitions of the [100] variant for the four unit cells with shearable precipitates. The results show that smaller edge lengths lead to stronger strain hardening, with a maximum stress difference of about 100 MPa at 5% applied strain. The length-scale effect on the stress partition of the [100] variant is minimal, with a maximum difference of only around 0.2%.

Figure 9c,d compare the stress–strain curves and stress partition for the unit cell model containing elastic precipitates. The stress difference at 5% applied strain is approximately 100 MPa, while the stress partition difference is around 0.1%. These results highlight that stress variation is strongly dependent on length scale. The stress partition within the variant is primarily governed by dislocation slip.

**Figure 10:**
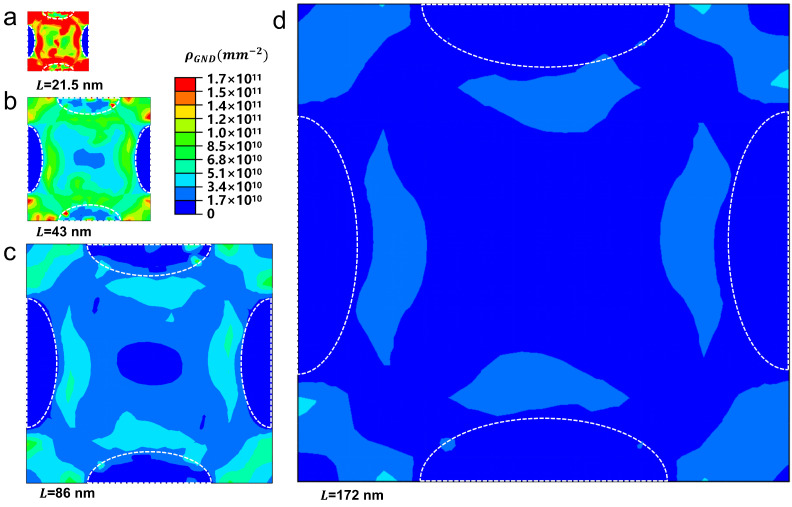
Strain gradient effect in terms of GND density distribution in the unit cell model with various side lengths: (**a**) 21.5 nm, (**b**) 43 nm, (**c**) 86 nm, and (**d**) 172 nm.

Figure 10 depicts the distribution of the GND density across four unit cell models at an applied strain of 5%. The results show that there is a tendency for the GND density to decline as the model size increases. This decrease in GND density subsequently results in a corresponding reduction in strain hardening. Moreover, within each model, it is evident that the matrix has a higher GND density than the precipitates, which is consistent with the findings of molecular dynamics (MD) simulations [46].

## 5. Discussion

Understanding anisotropy is crucial for improving the manufacturing technologies of the IN718 alloy, particularly in additive manufacturing and heat treatment processes. Two key structural factors known to influence the anisotropy of IN718 are polycrystalline texture and precipitate variant selection within the grain interior [42,66]. This study focuses on the interaction between dislocations and precipitate variants. This work indicates that the three precipitate variants interact differently with dislocation movements, resulting in significant strain and stress heterogeneities. These findings suggest that the IN718 superalloy exhibits strong anisotropy during plastic deformation, both at the precipitate level and the polycrystalline level. The presence of different variants can alter the distribution of stress and strain, stress partitioning, and the distribution of dislocation densities. The selection of precipitate variants, which can be induced thermo-mechanically during aging treatment [66], is therefore expected to play a critical role in governing nano-scale inelastic deformation behavior. Future work will delve into the roles of specific precipitate variants in contributing to the inelastic anisotropy of the alloy. Additionally, this study simplifies the uniaxial loading direction in the unit cell model to be parallel to the crystal axis of the matrix γ phase. Under other uniaxial loading directions or multiaxial loading conditions, the material’s constitutive response is expected to be heavily influenced by the $\gamma^{\prime \prime }$ precipitate variants. Detailed examination of this aspect will be another avenue of future research, which could lead to the development of a new constitutive law for the anisotropic IN718 single crystal.

Controlling the physical and geometrical features of precipitates to achieve desired strengthening properties is crucial for designing and engineering advanced metallic alloys. This paper has investigated the strain hardening behavior induced by the $\gamma^{\prime \prime }$ precipitate in the IN718 superalloy, with a particular focus on dislocation shearing and bypassing, while accounting for strain gradient effects. The study reveals that, on one hand, the $\gamma^{\prime \prime }$ precipitate exhibits relatively high slip resistance, impeding the movement of dislocations as they shear into the precipitate from the matrix. On the other hand, the nano-sized precipitates also obstruct dislocation motion within the matrix, leading to dislocation bypassing on certain slip planes. These two mechanisms—dislocation shearing and dislocation bypassing—are the primary factors influencing the strain hardening behavior of the material.

Table 4 compares the simulated stresses at 5% applied strain for the unit cell under different strengthening conditions. The results indicate that the dislocation bypassing mechanism contributes more significantly to strain hardening than dislocation shearing, consistent with the conventional strengthening mechanism map for these processes (e.g., [67]). Furthermore, incorporating the effect of the strain gradient near the interface between the $\gamma^{\prime \prime }$ precipitates and the γ matrix leads to an increase in overall stress of approximately 100 MPa for both cases of strengthening. This observation is consistent with previous literature (e.g., [33,53,68,69]), which highlights the predominant role of GND-associated strain gradients at the nanoscale in strain hardening.

However, this work is not without limitations. One significant flaw lies in the implementation of the strain gradient theory at the nanolevel. It is worth noting that there exists a non-uniqueness issue in determining GND density [70]. Optimization methods, such as minimizing stored strain energy or dislocation length and approximately solving geometrical constraint equations by minimizing residual normals, are commonly employed. However, the determination of GND density in the present work differs slightly from these methods. Here, the rate form of Nye’s closure failure equation in the initial configuration [71] is utilized. The associated uniqueness issue of the GND density rate is addressed by assuming that the generation of GND density in each slip system is independent of the slip behavior in other slip systems. This implementation has led to uncontrolled stress calculation results and an unrealistic GND density. The calculated GND density appears to be excessively high. However, GNDs emerge as an inherent component of the iterative solution process, serving to satisfy the governing equations of the model under the defined specific boundary conditions and material behavior assumptions. The principal objective of this study was to precisely calculate the size effect on stress–strain data, and the GND-related calculations are indispensable to this investigation. By delving into the implications of the GND-induced strain gradient theory within a small-scale context, we posit that our work makes a significant contribution by exploring the applicable limits of this theory.

In the realm of time scales, when compared with MD simulations, the proposed model is only applicable to low strain rates. Beyond this range, the model fails to fully account for the influence of the size effect on yield stress. It posits a fixed initial shear stress that is unrelated to precipitate size. As a result, the model merely considers how the size effect impacts stress flow, which is achieved through the evolution of both SSD and GND. Given that precipitates play a pivotal role in determining the yield stress of alloys like IN718, this omission severely limits the model’s predictive accuracy and practical applicability. Future research will focus on addressing these limitations in this area.

In addition, the anisotropic behavior of the IN718 alloy, which is significantly influenced by the $\gamma^{\prime \prime }$ phase, has not been addressed in this study. Future work should focus on exploring this aspect in detail. Understanding the anisotropic behavior stemming from the $\gamma^{\prime \prime }$ phase will be essential for a more complete and accurate description of IN718’s mechanical response, especially in applications where directional properties are critical.

## 6. Concluding Remarks

The main results and findings of this work can be drawn as follows:

1. The length-scale dependent crystal plasticity modeling results reveal distinct behaviors of the three $\gamma^{\prime \prime }$ variants, which significantly influence the deformation process in the γ phase and lead to pronounced strain and stress heterogeneity. Specifically, the [100] and [001] variants exhibit higher stress partitioning compared with the [010] variant under uniaxial [010] tensile loading, enabling them to accommodate dislocation shearing more effectively. This behavior reduces strain localization and dislocation density in the adjacent γ phase, highlighting the critical role of variant-specific interactions in governing deformation mechanisms.
2. During plastic deformation, the dislocation density, especially at phase boundaries, shows a notable increase. Through the CPFE model, the length scale effect was systematically measured. The results quantitatively indicate that a reduction in $\gamma^{\prime \prime }$ precipitate size corresponds to a significant enhancement in strain hardening. For example, a $\gamma^{\prime \prime }$ precipitate significantly influences the evolution of the GND density, although a relatively weak length-scale effect was observed on the stress partitioning among the three precipitate variants. The flow stress difference between the largest and smallest size of the precipitate currently is about 100 MPa at 5% applied strain.
3. This study provides critical information on the micromechanical behavior of $\gamma^{\prime \prime }$ precipitates in nickel-based superalloys, particularly IN718, elucidating the interaction between precipitate size, variant-specific deformation, and strain hardening. The findings underscore the importance of tailoring precipitate morphology and distribution to optimize mechanical performance in high-temperature applications. These results offer practical guidance for the design and processing of advanced alloys, enabling improved control over their deformation and strengthening mechanisms under service conditions.

## Figures and Tables

**Table 1:** Nominal chemical composition of IN718 (wt%).

Ni	Cr	Fe	Nb	Mo	Ti	Al	C
Balance	18.05	18.00	5.42	2.90	0.91	0.48	0.02

**Table 2:** Elastic constants, plastic flow, and strengthening parameters in the polycrystal FE model.

Elastic Constants	Flow Rule	Hardening Laws
$C_{11}$ = 235.5 GPa	$\dot{\gamma_{0}}=450$ $s^{-1}$	$h_{0}=0.0001$, b=0.254 nm
$C_{12}$ = 153.1 GPa	p=0.5	$K_{e}=0.6$, $K_{s}=2K_{e}$
$C_{44}$ = 125.4 GPa	q=1.25	$d_{e}=0.508$ nm, $d_{s}=10d_{e}$
$\mu =C_{44}$	F=286 kJ/mol	$\omega_{1S}=\omega_{2S}=1$
	$\tau_{0}=700$ MPa	$\omega_{1G}=\omega_{2G}=0$

**Table 3:** Phase-dependent parameters in the FE models at different scales.

	$\gamma^{\prime \prime }$ Phase	γ Phase	Homogenized Crystal
$K_{e}$	0.05	0.05	0.6
$h_{0}$	0.0004	0.0001	0.0001
$\tau_{0}$ (MPa)	900	700	700

**Table 4:** Comparison of the stress at ε=5% under different strengthening conditions.

Mechanisms	Shearing and Bypassing		Bypassing	
	With SG	Without SG	With SG	Without SG
Stress at ε=5% (MPa)	1243	1154	1342	1234

## Data Availability

The original contributions presented in this study are included in the article. Further inquiries can be directed to the corresponding author.

## Abbreviations

The following abbreviations are used in this manuscript:

IN718	Inconel 718
CP	Crystal plasticity
MD	Molecular dynamics
CRSS	Critical resolved shear stress
EBSD	Electron backscatter diffraction
FE	Finite element
CPFE	Crystal plasticity finite element
SSD	Statistically stored dislocation
GND	Geometrically necessary dislocation

## References

[1] Hall E. (1951). The deformation and ageing of mild steel: III discussion of results. Proc. Phys. Soc. Sect. B.

[2] Decker R. (2006). The evolution of wrought age-hardenable superalloys. JOM.

[3] Reed R.C. (2008). The Superalloys: Fundamentals and Applications.

[4] Nageswara Rao M. (2010). Factors influencing the notch rupture life of superalloy 718. Trans. Indian Inst. Met..

[5] Bridges D., Xu R., Hu A. (2019). Microstructure and mechanical properties of Ni nanoparticle-bonded Inconel 718. Mater. Des..

[6] Bai P., Huo P., Wang J., Yang C., Zhao Z., Zhang Z., Wang L., Du W., Qu H. (2022). Microstructural evolution and mechanical properties of Inconel 718 alloy manufactured by selective laser melting after solution and double aging treatments. J. Alloy. Compd..

[7] Holfelder P., Brenner F., Rund M., Witte A., Junghans S., Seyfert C., Richter M., Dell H., Koukolikova M., Gese H. (2022). Finite element simulation of plasticity and fracture for Inconel 718 deposited by laser powder bed fusion—Chances, use and challenges. Addit. Manuf..

[8] Oblak J., Paulonis D., Duvall D. (1974). Coherency strengthening in Ni base alloys hardened by DO22 *γ*′ precipitates. Metall. Trans..

[9] Han Y.f., Deb P., Chaturvedi M. (1982). Coarsening behaviour of *γ*″-and *γ*′-particles in Inconel alloy 718. Met. Sci..

[10] Sundararaman M., Mukhopadhyay P., Banerjee S. (1988). Deformation behaviour of *γ*″ strengthened inconel 718. Acta Metall..

[11] Qin H., Bi Z., Yu H., Feng G., Zhang R., Guo X., Chi H., Du J., Zhang J. (2018). Assessment of the stress-oriented precipitation hardening designed by interior residual stress during ageing in IN718 superalloy. Mater. Sci. Eng. A.

[12] Zhang D., Guan Z., Qian W., Ye Y., Dai F., Hua Y., Cai J. (2023). The effect of heterogeneous precipitation behavior on the mechanical properties of IN718 superalloy after laser shock peening and heat treatment processes. J. Manuf. Process..

[13] Yuan G.J., Chen H., Li D.F., Gong C.Y., Zhang X.C., Tu S.T. (2020). The effect of *δ* phase on the microplasticity evolution of a heat-treated nickel base superalloy. Mech. Mater..

[14] Rielli V.V., Godor F., Gruber C., Stanojevic A., Oberwinkler B., Primig S. (2021). Effects of processing heterogeneities on the micro-to nanostructure strengthening mechanisms of an alloy 718 turbine disk. Mater. Des..

[15] Adomako N.K., Haghdadi N., Primig S. (2022). Electron and laser-based additive manufacturing of Ni-based superalloys: A review of heterogeneities in microstructure and mechanical properties. Mater. Des..

[16] Merrick H. (1974). The low cycle fatigue of three wrought nickel-base alloys. Metall. Trans..

[17] Chaturvedi M., Han Y.f. (1983). Strengthening mechanisms in Inconel 718 superalloy. Met. Sci..

[18] Fisk M., Ion J.C., Lindgren L.E. (2014). Flow stress model for IN718 accounting for evolution of strengthening precipitates during thermal treatment. Comput. Mater. Sci..

[19] McAllister D., Lv D., Peterson B., Deutchman H., Wang Y., Mills M. (2016). Lower temperature deformation mechanisms in a *γ*″-strengthened Ni-base superalloy. Scr. Mater..

[20] Eghtesad A., Knezevic M. (2021). A full-field crystal plasticity model including the effects of precipitates: Application to monotonic, load reversal, and low-cycle fatigue behavior of Inconel 718. Mater. Sci. Eng. A.

[21] Holmberg-Kasa J., Olsson P.A.T., Fisk M. (2024). Investigating Elastic Deformation of Ordered Precipitates by Ab Initio-Informed Phase-Field Crystal Modeling. Metals.

[22] Han Y., Chaturvedi M. (1987). A study of back stress during creep deformation of a superalloy inconel 718. Mater. Sci. Eng..

[23] Kurdi A., Aldoshan A., Alshabouna F., Alodadi A., Degnah A., Alnaser H., Tabbakh T., Basak A.K. (2023). Investigation into the Microstructure and Hardness of Additively Manufactured (3D-Printed) Inconel 718 Alloy. Materials.

[24] Meng G., Gong Y., Zhang J., Zhao J. (2024). The microstructural evolution and mechanical response of laser direct energy deposition Inconel 718 alloy based on simulation and experimental methods. Eng. Fail. Anal..

[25] Zhang S., Guo C., Lin X., Zhao H., Yang H., Huang W. (2024). Deformation behavior of selective laser-melted Inconel 718 superalloy. Mater. Charact..

[26] Bacon D.J., Osetsky Y.N. (2004). Hardening due to copper precipitates in *α*-iron studied by atomic-scale modelling. J. Nucl. Mater..

[27] Osetsky Y.N., Pharr G.M., Morris J.R. (2019). Two modes of screw dislocation glide in fcc single-phase concentrated alloys. Acta Mater..

[28] Antillon E., Woodward C., Rao S., Akdim B., Parthasarathy T. (2019). A molecular dynamics technique for determining energy landscapes as a dislocation percolates through a field of solutes. Acta Mater..

[29] Vaida A., Guénoléb J., Prakashc A., Korte-Kerzelb S., Bitzeka E. (2019). Atomistic Simulations of Basal Dislocations Interacting with Mg17Al12 Precipitates in Mg. Materialia.

[30] Li J., Chen H., Fang Q., Jiang C., Liu Y., Liaw P.K. (2020). Unraveling the dislocation–precipitate interactions in high-entropy alloys. Int. J. Plast..

[31] Esteban-Manzanares G., Alizadeh R., Papadimitriou I., Dickel D., Barrett C., LLorca J. (2020). Atomistic simulations of the interaction of basal dislocations with MgZn_2_ precipitates in Mg alloys. Mater. Sci. Eng. A.

[32] Li J., Fang Q., Liu B., Liu Y. (2016). The effects of pore and second-phase particle on the mechanical properties of machining copper matrix from molecular dynamic simulation. Appl. Surf. Sci..

[33] Meissonnier F., Busso E., O’Dowd N. (2001). Finite element implementation of a generalised non-local rate-dependent crystallographic formulation for finite strains. Int. J. Plast..

[34] Roters F., Eisenlohr P., Hantcherli L., Tjahjanto D.D., Bieler T.R., Raabe D. (2010). Overview of constitutive laws, kinematics, homogenization and multiscale methods in crystal plasticity finite-element modeling: Theory, experiments, applications. Acta Mater..

[35] Ghorbanpour S., Zecevic M., Kumar A., Jahedi M., Bicknell J., Jorgensen L., Beyerlein I.J., Knezevic M. (2017). A crystal plasticity model incorporating the effects of precipitates in superalloys: Application to tensile, compressive, and cyclic deformation of Inconel 718. Int. J. Plast..

[36] Cruzado A., Lucarini S., LLorca J., Segurado J. (2018). Crystal plasticity simulation of the effect of grain size on the fatigue behavior of polycrystalline Inconel 718. Int. J. Fatigue.

[37] Agaram S., Kanjarla A.K., Bhuvaraghan B., Srinivasan S.M. (2021). Dislocation density based crystal plasticity model incorporating the effect of precipitates in IN718 under monotonic and cyclic deformation. Int. J. Plast..

[38] le Graverend J.B. (2023). Crystal-plasticity modeling of monotonic and cyclic softening in inconel 718 superalloy. Int. J. Mech. Sci..

[39] Lai R., Zhao J., Lei L., Shi L., Wu S., Zhang X. (2025). Revealing the tensile anisotropic mechanisms of additive manufactured IN718 alloy based on crystal plasticity modeling. Comput. Mater. Sci..

[40] Zhu K.Y., Dai S., Zou S.H., Yu Y.J., Deng Z.C. (2024). Experimental study and crystal plasticity modeling of additive manufacturing IN718 superalloy considering negative strain rate sensitivity behavior. Eur. J. Mech.—A/Solids.

[41] Knezevic M., Ghorbanpour S. (2018). Modeling Tensile, Compressive, and Cyclic Response of Inconel 718 Using a Crystal Plasticity Model Incorporating the Effects of Precipitates. Proceedings of the 9th International Symposium on Superalloy 718 & Derivatives: Energy, Aerospace, and Industrial Applications.

[42] Ghorbanpour S., Alam M.E., Ferreri N.C., Kumar A., McWilliams B.A., Vogel S.C., Bicknell J., Beyerlein I.J., Knezevic M. (2020). Experimental characterization and crystal plasticity modeling of anisotropy, tension-compression asymmetry, and texture evolution of additively manufactured Inconel 718 at room and elevated temperatures. Int. J. Plast..

[43] Li D.F., Golden B.J., O’Dowd N.P. (2014). Multiscale modelling of mechanical response in a martensitic steel: A micromechanical and length-scale-dependent framework for precipitate hardening. Acta Mater..

[44] Jiang W., Xu P., Li Y., Wang H., Cai Z., Li J., Liang Y., Liang Y. (2023). Effect of a gradient structure on the mechanical performance of Inconel 718 Ni-based superalloy at elevated temperatures. J. Mater. Res. Technol..

[45] Toursangsaraki M., Du D., Wang H., Dong A. (2024). Crystal plasticity quantification of anisotropic tensile and fatigue properties in laser powder bed fused Inconel 718 superalloy. Addit. Manuf..

[46] Wan C.F., Sun L.G., Qin H.L., Bi Z.N., Li D.F. (2023). A Molecular Dynamics Study on the Dislocation-Precipitate Interaction in a Nickel Based Superalloy during the Tensile Deformation. Materials.

[47] Russakova A., Zhilkashinova A., Alontseva D., Abilev M., Khozhanov A., Zhilkashinova A. (2023). Effect of the Dislocation Substructure Parameters of Hadfield Steel on Its Strain Hardening. Materials.

[48] Wu P., Song K., Liu F. (2024). Effect of Coherent Nanoprecipitate on Strain Hardening of Al Alloys: Breaking through the Strength-Ductility Trade-Off. Materials.

[49] Jinhui D., Xudong L., Qun D., Ying L. (2017). Effect of solution treatment on the microstructure and mechanical properties of IN718 alloy. Rare Met. Mater. Eng..

[50] Qin H., Bi Z., Li D., Zhang R., Lee T.L., Feng G., Dong H., Du J., Zhang J. (2019). Study of precipitation-assisted stress relaxation and creep behavior during the ageing of a nickel-iron superalloy. Mater. Sci. Eng. A.

[51] Li D.F., Barrett R.A., O’Donoghue P.E., O’Dowd N.P., Leen S.B. (2017). A multi-scale crystal plasticity model for cyclic plasticity and low-cycle fatigue in a precipitate-strengthened steel at elevated temperature. J. Mech. Phys. Solids.

[52] Lee E.H. (1969). Elastic-plastic deformation at finite strains. J. Appl. Mech..

[53] Asaro R.J., Rice J. (1977). Strain localization in ductile single crystals. J. Mech. Phys. Solids.

[54] Li D.F., O’Dowd N.P. (2011). On the evolution of lattice deformation in austenitic stainless steels—The role of work hardening at finite strains. J. Mech. Phys. Solids.

[55] Busso E.P., McClintock F.A. (1996). A dislocation mechanics-based crystallographic model of a B2-type intermetallic alloy. Int. J. Plast..

[56] Cheong K., Busso E., Arsenlis A. (2005). A study of microstructural length scale effects on the behaviour of FCC polycrystals using strain gradient concepts. Int. J. Plast..

[57] Zhang R., Qin H., Bi Z., Li J., Paul S., Lee T., Nenchev B., Zhang J., Kabra S., Kelleher J. (2019). Using variant selection to facilitate accurate fitting of *γ*″ peaks in neutron diffraction. Metall. Mater. Trans. A.

[58] Gnäupel-Herold T., Brand P.C., Prask H.J. (1998). Calculation of single-crystal elastic constants for cubic crystal symmetry from powder diffraction data. J. Appl. Crystallogr..

[59] Weinberger C.R., Boyce B.L., Battaile C.C. (2013). Slip planes in bcc transition metals. Int. Mater. Rev..

[60] Li D.F., Davies C.M., Zhang S.Y., Dickinson C., O’Dowd N.P. (2013). The effect of prior deformation on subsequent microplasticity and damage evolution in an austenitic stainless steel at elevated temperature. Acta Mater..

[61] Cruzado A., Gan B., Chang H., Ostolaza K., Linaza A., Milenkovic S., Molina-Aldareguia J., Llorca J., Segurado J. Microtesting and Crystal Plasticity Modelling of IN718 Superalloy Grains. Proceedings of the 8th International Symposium on Superalloy.

[62] Cheong K.S., Busso E.P. (2006). Effects of lattice misorientations on strain heterogeneities in FCC polycrystals. J. Mech. Phys. Solids.

[63] Society C.M. (2012). China Superalloys Handbook.

[64] Orowan E. (1949). Fracture and strength of solids. Rep. Prog. Phys..

[65] Voyiadjis G., Yaghoobi M. (2019). Size Effects in Plasticity: From Macro to Nano.

[66] Qin H., Bi Z., Zhang R., Lee T.L., Yu H., Chi H., Li D., Dong H., Du J., Zhang J. (2020). Stress-Induced Variant Selection of *γ*″ Phase in Inconel 718 During Service: Mechanism and Effects on Mechanical Behavior. Proceedings of the 14th International Symposium on Superalloys (Superalloys 2020).

[67] Lv D., McAllister D., Mills M., Wang Y. (2016). Deformation mechanisms of D022 ordered intermetallic phase in superalloys. Acta Mater..

[68] Ashby M. (1970). The deformation of plastically non-homogeneous materials. Philos. Mag. A J. Theor. Exp. Appl. Phys..

[69] Hughes D., Hansen N., Bammann D. (2003). Geometrically necessary boundaries, incidental dislocation boundaries and geometrically necessary dislocations. Scr. Mater..

[70] Dunne F., Kiwanuka R., Wilkinson A. (2012). Crystal plasticity analysis of micro-deformation, lattice rotation and geometrically necessary dislocation density. Proc. R. Soc. A Math. Phys. Eng. Sci..

[71] Busso E., Meissonnier F., O’dowd N. (2000). Gradient-dependent deformation of two-phase single crystals. J. Mech. Phys. Solids.

